# Combined scanning small-angle X-ray scattering and holography probes multiple length scales in cell nuclei

**DOI:** 10.1107/S1600577520016276

**Published:** 2021-01-21

**Authors:** Andrew Wittmeier, Chiara Cassini, Mareike Töpperwien, Manuela Denz, Johannes Hagemann, Markus Osterhoff, Tim Salditt, Sarah Köster

**Affiliations:** a Institute for X-Ray Physics, University of Göttingen, Friedrich-Hund-Platz 1, 37077 Göttingen, Germany; b Cluster of Excellence ‘Multiscale Bioimaging: from Molecular Machines to Networks of Excitable Cells (MBExC)’, University of Göttingen, Göttingen, Germany

**Keywords:** scanning small-angle X-ray scattering, X-ray holography, cell nucleus, multi-scale imaging, chromatin

## Abstract

X-rays are emerging as a complementary probe to visible-light photons and electrons for imaging biological cells. By exploiting their small wavelength and high penetration depth, it is possible to image whole, intact cells and resolve subcellular structures at nanometer resolution. A variety of X-ray methods for cell imaging have been devised for probing different properties of biological matter, opening up various opportunities for fully exploiting different views of the same sample. Here, a combined approach is employed to study cell nuclei of NIH-3T3 fibroblasts. Scanning small-angle X-ray scattering is combined with X-ray holography to quantify length scales, aggregation state, and projected electron and mass densities of the nuclear material. Only by joining all this information is it possible to spatially localize nucleoli, heterochromatin and euchromatin, and physically characterize them. It is thus shown that for complex biological systems, like the cell nucleus, combined imaging approaches are highly valuable.

## Abstract 2

The combination of small-angle X-ray scattering and X-ray holography enables us to visualize and characterize biological material in cell nuclei spanning multiple length scales.

## 1. Introduction   

DNA within mammalian cell nuclei stores genetic information and is densely packed. Indeed, about 2 m of DNA (Maeshima *et al.*, 2010 ▸) are found in the nucleus of each human cell within a diameter of roughly 10 µm. The packing hierarchy spans length scales from 2 nm to 1 µm. Techniques to image structures on these length scales primarily use three types of probes, *i.e.*, electrons (Koster & Klumperman, 2003 ▸; Lučić *et al.*, 2005 ▸), visible-light fluorescence (Stelzer *et al.*, 1991 ▸; Sahl *et al.*, 2017 ▸) and X-rays (Kirz *et al.*, 1995 ▸; Hémonnot & Köster, 2017 ▸). Electron microscopy (EM) has the highest spatial resolution but is very invasive, typically requiring the sample to be sliced and stained. Using EM, purified nucleofilaments with a diameter of 10 nm, which fold into higher-order fibers with a 30 nm diameter, were resolved (Finch & Klug, 1976 ▸), and it was shown that these fibers subsequently coil into a zigzag ribbon structure (Woodcock *et al.*, 1984 ▸).

Specific labeling of cellular components, as is employed in fluorescence microscopy, provides a straightforward way of identifying exactly these labeled components. Using a combination of fluorescence microscopy and electron tomography, the *in situ* 3D packing of chromatin in human mitotic chromosomes was described as a disordered and flexible granular chain (Ou *et al.*, 2017 ▸). With the invention of super-resolution fluorescence techniques (Lakadamyali & Cosma, 2015 ▸), *e.g.*, stimulated emission depletion (STED) microscopy (Hell & Wichmann, 1994 ▸), stochastic optical reconstruction microscopy (STORM) (Rust *et al.*, 2006 ▸), fluorescence-lifetime imaging microscopy (FLIM) (Lakowicz *et al.*, 1992 ▸) or Förster resonance energy transfer (FRET) microscopy (Förster, 1948 ▸), resolution of tens of nanometers can be achieved (Rust *et al.*, 2006 ▸; Lakadamyali & Cosma, 2015 ▸). Employing these innovative methods, small loops of DNA within mitotic chromosomes have been imaged (Spahn *et al.*, 2018 ▸) by STED microscopy and, using STORM, the structure of chromatin fiber was visualized *via* imaging the histone protein H2B (Ricci *et al.*, 2015 ▸). Within HeLa cells, the *in situ* interaction of the heterochromatin protein HP1α and DNA was imaged using a combination of FLIM and FRET microscopy (Cremazy *et al.*, 2005 ▸).

The strength of fluorescence microscopy is that cellular components are specifically labeled. However, as a consequence, only labeled structures can be imaged. By contrast, X-rays probe electron density directly and thus do not rely on labeling or staining. Moreover, the high penetration depth and small wavelength of X-rays allows us to image thick samples, like whole cells, with resolutions below 100 nm. Scanning small-angle X-ray scattering (SAXS) (Fratzl *et al.*, 1997 ▸), in particular, combines information from real space and Fourier space. The method was introduced in 1997 and was first used to resolve the size and orientation of particles embedded in bone collagen and cellulose fibrils in wood. Scanning SAXS was also successfully used to study teeth (Deyhle *et al.*, 2011 ▸), hair (Stanić *et al.*, 2015 ▸) and muscle tissue (Bunk *et al.*, 2009 ▸). More recently, scanning SAXS was applied to single cells (Weinhausen *et al.*, 2012 ▸) and, of particular interest for this present study, cell nuclei (Hémonnot *et al.*, 2016 ▸), where the aggregation and (de-)compaction of chromatin was followed throughout the cell cycle.

Another technique to obtain nanometer resolution utilizing X-rays is holography, a full-field propagation based near-field imaging approach. Quantitative phase contrast imaging is performed and the projected electron density, and thereby mass density, of the sample is investigated. Examples of successful application to single cells are *Bacillus thuringiensis* (Wilke *et al.*, 2015 ▸) and *Deinococcus radiodurans* (Bartels *et al.*, 2012 ▸, 2015 ▸), where resolutions of 100 nm, 53 nm and 125 nm, respectively, were obtained. The latter two studies are particularly interesting in the context of the present work as they focus on densely packed DNA in *Deinococcus radiodurans*.

Joining scanning SAXS and X-ray holography in the same experiment combines the strengths of both methods. Scanning SAXS probes structural and morphological information whereas X-ray holography provides quantitative electron and mass density. Combining these methods, Nicolas *et al.* (2017 ▸) were able to probe the orientation of actomyosin filaments within lyophilized neo-natal rat muscle cells and structural information spanning three orders of magnitude. This work was extended by correlating the X-ray holography and scanning SAXS data with STED images (Bernhardt *et al.*, 2018 ▸).

Here, we apply the very successful combination of scanning SAXS and X-ray holography to nuclei of mammalian cells, a biological system for which the hierarchical combination of different length scales fundamentally defines function. We extend the approach presented by Nicolas *et al.* (2017 ▸) and Bernhardt *et al.* (2018 ▸) by carefully and quantitatively analyzing four different physical quantities accessible by the combination of scanning SAXS and X-ray holography, namely the relevant length scales, morphology, aggregation and mass or electron density of the scatters, in a spatially resolved manner. We show that only by combining all of this information are we able to identify and localize important nuclear structures, *i.e.*, nucleoli, heterochromatin and euchromatin, thus highlighting the relevance of combined imaging, and characterize the structures according to size, aggregation and density. Thus, we present the results of a label-free technique that is widely applicable to biological samples and can spatially distinguish scattering biological matter across various length scales.

## 2. Materials and methods   

### 2.1. Sample preparation   

NIH-3T3 fibroblasts derived from Swiss albino mouse embryos (Todaro & Green, 1963 ▸) were cultured in cell culture flasks (Nunc A/S, Roskilde, Denmark) with a 25 cm^2^ area using high-glucose (4.5 g L^−1^) Dulbecco’s Modified Eagle’s Medium (Sigma-Aldrich, Taufkirchen, Germany) supplemented with 10% (v/v) FBS (Sigma) and 1% (v/v) penicillin-streptomycin. The cells were stored in a cell incubator kept at 37°C and 5% CO_2_. Once the cells reached a confluency of ∼80% they were detached from the flask *via* trypsin incubation (37°C, 5% CO_2_) for 150 s using 0.05% trypsin derived from porcine pancreas (Sigma-Aldrich). A silicon-rich nitride membrane (Si_3_N_4_; membrane size and thickness: 1.5 mm × 1.5 mm and 1 µm; frame size and thickness: 5.0 mm × 5.0 mm and 200 µm; Silson Ltd, Warwickshire, UK), which was previously plasma cleaned using a radiofrequency power of 18 W for 30 s (Harrick Plasma, PDC-32G, Ithaca, New York, USA), was placed in a 3 cm-diameter Petri dish, where 1.7 ml of medium and 300 µL of cell suspension, with approximately 3.8  × 10^5^ cells ml^−1^, were subsequently added. The Petri dish was then placed into the incubator for roughly 48 h to promote cell adhesion to the Si_3_N_4_ membrane. The cells were chemically fixed using 3.7% formaldehyde, stabilized with 1% methanol, and were then rinsed with (1×) phosphate buffered saline (Sigma-Aldrich). After the chemical fixation process the cells were vitrified by rapidly plunging them from a water-saturated environment (humidity ≥ 95%, 19°C) into a liquid ethane/propane bath at −196°C using a Leica grid plunger (Leica EM GP, Leica Microsystems, Wetzlar, Germany). The cells were stored in liquid nitrogen until the lyophilization process began, where they were transferred into a home-built evacuated chamber which was kept in cryogenic conditions. The temperature of the lyophilization process was gradually increased from −186°C to 15°C. The cells were kept inside the chamber, with a pressure on the order of 0.01 Pa, for several days in order to ensure the sublimation of any amorphous ice formed during the plunging process. After the lyophilization process, the cell thickness is approximately 3 µm. To prevent rehydration, the cells were kept in an evacuated desiccator until they were mounted on the sample stage at the beamline. An inverted light microscope (IX81, Olympus, Hamburg, Germany) was used to record phase contrast micrographs (20× objective) immediately before and after the plunging and lyophilization steps; these images were used to monitor the sample preparation process.

### 2.2. Experimental setups   

**Figure 1:**
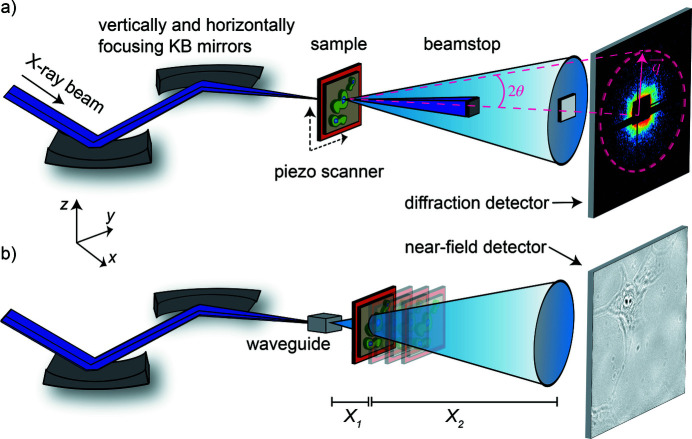
Schematics of the setups used for (*a*) scanning SAXS and (*b*) in-line holography using the GINIX endstation at the P10 coherence application beamline at the PETRA III storage ring, DESY, Hamburg (Kalbfleisch *et al.*, 2011 ▸; Salditt *et al.*, 2015 ▸). (*a*) The X-rays are focused by a set of Kirkpatrick–Baez (KB) mirrors prior to being scattered from the sample. The sample is raster scanned in the *y*–*z* plane and at each scanning position a scattering pattern is recorded by a single-photon-counting pixelated detector. The dashed purple lines illustrate the relationship between the scattering angle 2θ and the momentum transfer vector **q**. (*b*) The KB-focused beam is coupled into a waveguide which acts as a quasi point-source creating a diverging wavefront. The sample is placed at a series of defocused positions *X*_1_ and full-field holograms are recorded at each distance with a sCMOS imaging camera located at a distance *X*_2_ behind the sample.

All measurements detailed in this work were performed using the Göttingen Instrument for Nano-Imaging with X-rays (GINIX) (Kalbfleisch *et al.*, 2011 ▸; Salditt *et al.*, 2015 ▸) end­station at the coherence applications beamline P10 at the PETRA III storage ring (DESY, Hamburg, Germany). The beam was delivered *via* a 5 m-long undulator and subsequently monochromatized by a Si-111 double-crystal monochromator to an energy of 8.0 keV. Entrance slits to the Kirkpatrick–Baez (KB) mirrors (Kirkpatrick & Baez, 1948 ▸) were tuned to 0.4 mm × 0.4 mm. The KB mirrors, oriented in a cross-orthogonal manner, focused the beam to a spot size of 350 nm × 390 nm (vertical × horizontal, FWHM). The beam had an intensity of approximately 5.5 × 10^11^ photons s^−1^ and was cleaned by apertures prior to interacting with the sample, which was mounted on a piezo-electric stage capable of lateral movement with nanometer precision. An on-axis visible-light microscope, operated in reflectivity mode, was used to locate a desired scanning region of the sample. Scanning SAXS measurements were performed by continuously moving the sample horizontally (200 steps) and vertically (200 steps) through the X-ray beam in steps of 250 nm. Thus, a total of 201 × 201 = 40401 diffraction patterns were recorded. With an exposure time of 10 ms, the entire scan took about 14 min to complete, including about 7 min of overhead due to data transfer. Note that during the overhead time the sample region was moved out of the beam path and no impact of photons occurred. Behind the sample, the X-rays propagated through a 5 m-long evacuated flight tube where the primary beam was blocked by a tungsten beamstop (size: 7 mm × 8 mm; thickness: 25 µm). The scattering signal was recorded using a single-photon-counting detector (Eiger 4M, Dectris AG, Baden-Dättwil, Switzerland; 2070 × 2167 pixels; pixel size: 75 µm × 75 µm). The effective pixel size of the scanning SAXS measurements was equal to the step size, and the field of view was 50 µm × 50 µm. A schematic of the scanning SAXS setup is shown in Fig. 1(*a*) ▸.

An attractive feature of the GINIX endstation is its ability to change between the scanning SAXS and in-line holography imaging modalities with only minor hardware reconfigurations (Salditt *et al.*, 2015 ▸). To this end, the sample was (initially) placed 25 mm downstream from the KB focal plane and the focused beam was coupled into an X-ray waveguide consisting of lithography-defined channels in silicon (Chen *et al.*, 2015 ▸). The waveguide not only coherently filtered the beam but also acted as a quasi point-source (≤20 nm), producing spherical wavefronts. After coupling into the waveguide, the transmitted intensity of the beam was approximately 2.9 × 10^9^ photons s^−1^. In the sample plane individual holograms had a field of view (FOV) of 33 µm × 33 µm and were recorded by a scientific CMOS (sCMOS) camera (Photonic Science, East Sussex, UK; 2048 × 2048 pixels; pixel size: 6.5 µm × 6.5 µm) located on the same detector bench as the Eiger 4M detector. Multiple holograms could be imaged in a mosaic fashion to accommodate a larger FOV. Holograms were acquired at distances of *X*_1_ = {24.9, 26.9, 31.8, 38.5} mm, with respect to the focus position at *X*_0_ = 0 mm, with corresponding magnifications and effective pixel sizes of {201.8, 186.9, 157.7, 130} and {32.4, 34.9, 41.5, 50.3} nm, respectively. Per distance, 10 images were acquired with an exposure time of 60 ms each. Additionally, 50 empty images were taken per distance. An empty image is the recorded intensity when the sample is not in the beam path. A schematic of the holographic imaging setup is shown in Fig. 1 ▸(*b*).

The radiation dose differs between the two imaging modalities by several orders of magnitude. The dose is estimated from the intensity *I*_0_ and energy *E*_ph_ of the incident beam, as well as the exposure time τ and irradiated area Δ_*y*_Δ_*z*_,
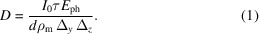
For calculating the dose on a biological sample with an averaged empirical formula H_50_C_30_N_9_O_10_S_1_, as is considered here, an attenuation length of *d* = 7.5 × 10^−4^ m and mass density of 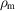 = 1.35 g cm^−3^ are commonly used (Howells *et al.*, 2009 ▸; Shen *et al.*, 2004 ▸). Substituting the appropriate values into equation (1), a dose of 1.1 × 10^8^ Gy and 8.6 × 10^3^ Gy was calculated for the scanning SAXS and X-ray holography measurements, respectively. For the holography calculation, the value of τ represents the accumulation of the individual exposure times at each of the four defocused positions. Furthermore, an additional 50 ms was considered per distance to account for the time required to open and close the beam shutter. For the scanning SAXS calculation only the exposure time and no overhead time was considered as the scanning ROI itself was not exposed to the X-ray beam during data transfer. In order to preserve the sample’s structural integrity as well as possible, holography measurements were performed prior to the more invasive scanning SAXS measurements. In fact, related experiments indicate considerable beam damage during scanning SAXS measurements (Weinhausen *et al.*, 2012 ▸; Nicolas *et al.*, 2017 ▸).

### 2.3. Data analysis   

**Figure 2:**
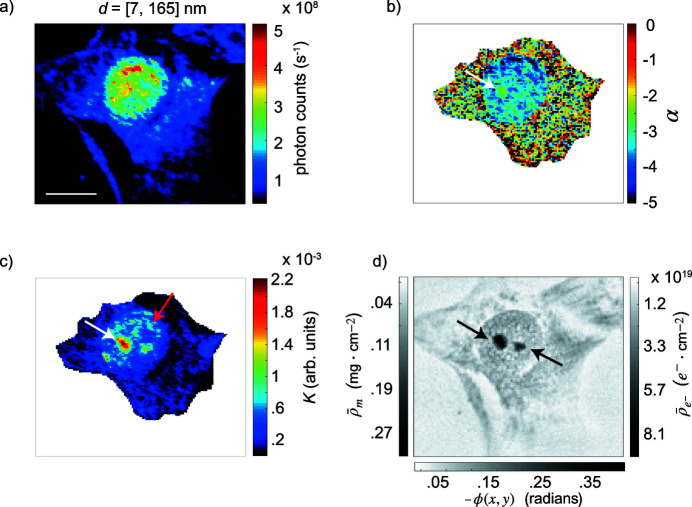
(*a*) X-ray dark field image of a typical NIH-3T3 fibroblast; here, the full data range of [*q*_0_, *q*_max_] = [0.038, 0.867] nm^−1^ is taken into account for calculating the dark field image. The color bar represents the total number of scattered photons per second. (*b*) Map of the exponent α for partial fitting ranges; [*q*_0_, *q*_min_] = [0.038, 0.132] nm^−1^, [0.038, 0.085] nm^−1^ and [0.038, 0.052] nm^−1^ for the heterochromatin, euchromatin and cytoplasm ROIs, respectively. See Fig. 4 ▸(*a*) for the real-space ROI definitions. (*c*) Map of the Porod constant *K* for partial fitting ranges; [*q*_min_, *q*_max_] = [0.132, 0.867] nm^−1^, [0.085, 0.867] nm^−1^ and [0.052, 0.867] nm^−1^ for the heterochromatin, euchromatin and cytoplasm, respectively. For both (*b*) and (*c*), the values are derived by fitting radial intensity profiles corresponding to individual scattering patterns. (*d*) The reconstructed phase map, rendered by reconstructing holograms recorded at a single defocused position using the RAAR (Luke, 2005 ▸) algorithm. The 2D projected mass and electron densities are also shown (grayscale bars on the left- and right-hand side, respectively). The scale bar in (*a*) is 10 µm and applies to all subfigures.

The recorded intensities of the scanning SAXS and X-ray holography measurements correspond to the (Fraunhofer) far-field and (Fresnel) near-field, respectively. For the scanning SAXS measurements, to first visualize the cell in a pseudo real space representation, every 2D scattering pattern was multiplied by a logic mask which rendered unwanted regions (beamstop, flight tube, dead pixels) null from subsequent analysis. By integrating the remaining number of scattered photons of each scattering pattern and plotting the resulting value in a color-coded fashion at its corresponding scanning location, we obtain an X-ray dark field image. An example of a dark field image is shown in Fig. 2 ▸(*a*) and cellular and background regions of interest (ROIs) were manually defined (Fig. 4*a*). Averaged and individual scattering patterns belonging to each ROI were investigated throughout this work. Full 2D scattering patterns were azimuthally integrated and plotted against the magnitude of the momentum transfer wavevector *q* to obtain 1D radial intensity profiles *I*(*q*). The values of *q* are related to the scattering angle 2θ and X-ray wavelength λ (Porod, 1951 ▸; Guinier & Fournet, 1955 ▸; Glatter & Kratky, 1982 ▸) *via*

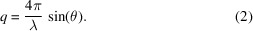
This relationship is schematically shown in Fig. 1 ▸(*a*). Due to the sizes of the beamstop and detector modules, the low and high spatial frequencies were limited to *q*_0_ = 0.038 nm^−1^ and *q*_max_ = 0.867 nm^−1^, respectively, corresponding to a range of [7, 165] nm in real space. The radial intensity profile *I*(*q*) of each ROI was background-corrected by subtraction of the *I*(*q*) profile corresponding to the average diffraction pattern of the background ROI (see Fig. S1 in the supporting information). The background-corrected *I*(*q*) curves were then normalized by the exposure time and fitted with a power law [equation (3)] using a non-linear least-squares minimization. When fitting the *I*(*q*) profiles, we took into account the error associated with the azimuthal integration, 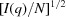, with the number of pixels *N* along the circumference. The fitting procedure was performed twice: once between *q*_0_ and *q*_min_ to determine the exponent α, and a second time between *q*_min_ and *q*_max_ to determine *K*. The momentum transfer *q*_min_ denotes the point in an *I*(*q*) profile where the slope transitions from ≠−4 to −4. The [*q*_min_, *q*_max_] range was uniquely determined for each of the ROIs of every individual cell using the following criteria: every possible *q*-range of an averaged *I*(*q*) profile was fitted using equation (3) and we chose the threshold to require the difference between the fit coefficient for α and the theoretical value α = −4 to be less than one standard error. The largest *q*-range which satisfied the threshold was subsequently used to define [*q*_min_, *q*_max_] for the particular ROI of the individual cell. This *q*-range defined the fitting range used when fitting individual *I*(*q*) profiles to determine the Porod constant *K*.

Concerning the holography measurements, the acquired holograms of each distance were first averaged and background corrected by division by their respective averaged empty image. The background corrected holograms were subsequently rescaled to match the magnification of those recorded at the first defocused position and were then aligned *via* a sub-pixel image registration algorithm (Guizar-Sicairos *et al.*, 2008 ▸) to account for any lateral shifts that occurred during the image acquisition process. Following these steps, the in-line holograms were ready for both direct and iterative reconstruction algorithms. To this end, the holograms were first numerically processed using the contrast transfer function (CFT) algorithm (Zabler *et al.*, 2005 ▸). The resulting 2D reconstructed phase map was used to define the support constraint for the *single distance* iterative relaxed averaged alternating reflections (RAAR) (Luke, 2005 ▸) algorithm. In total, 500 iteration rounds were performed to render the reconstructed phase map observed in Fig. 2 ▸(*d*). The reconstructed phase contrast, ϕ(*x*, *y*) = ϕ_sample_ − ϕ_bg_, was used to quantify both projected electron density [equation (5)] and projected mass density [equation (6)].

The illuminated areas of the sample differ in the scanning SAXS and holography measurements. Consequently, the FOV and effective pixel sizes rendered from each modality differ. To account for this circumstance, and to correlate a reconstructed phase map, and subsequently a projected mass or electron density map, to those derived from the SAXS measurements in a pixel-wise manner, image registration is performed. To this end, a reconstructed phase map was registered to a dark field image *via* a self-written MATLAB (The MathWorks, Inc., Natick, MA, USA) script. The reconstructed phase map was first resized such that it had the same effective pixel size as that of the dark field. The *cpselect* tool was then used to manually select several anchor points. Any prominent features that were clearly distinguishable in both images, *e.g.*, nucleoli or the outline of the cell body, were suitable candidates for anchors. On average, seven anchors were defined per cell. Using the *fitgeotrans* command, the reconstructed phase map then underwent the necessary rotational, scaling and translational transforms such that its anchor points had the same spatial coordinates as those in the dark field.

## 3. Results and discussion   

### 3.1. Aggregation state of DNA in cell nuclei   

All measurements presented in this work are performed on lyophilized NIH-3T3 fibroblasts cultured on X-ray transmissive silicon-nitride substrates. Although in general the lyophilization process may alter nanostructures and thus damage the sample, Zhang *et al.* (2017 ▸) found that the integrity of mammalian DNA remains intact directly after the process. For scanning SAXS measurements, the sample is placed in the focal plane of the X-ray beam and is subsequently raster scanned, as depicted in Fig. 1 ▸(*a*). At each scanning position a scattering pattern, determined by the size, morphology and electron density of the scatterers, is recorded. For every cell investigated here, 40401 scattering patterns are recorded and used to render dark field images. The dark field contrast provides an integrated quantity that does not distinguish between the length scales covered by the recorded data range. A dark field is a pseudo real space representation of a sample and physically represents its overall granularity. An example for such a dark field image calculated using the entire usable data range [*q*_0_, *q*_max_] = [0.038, 0.867] nm^−1^, corresponding to length scales in the [7, 165] nm range in real space, of a cell in interphase is shown in Fig. 2 ▸(*a*). Visible-light micrographs of the cell in the chemically fixed and lyophilized state are shown in Figs. S2(*a*) and S2(*b*) in the supporting information.

**Figure 3:**
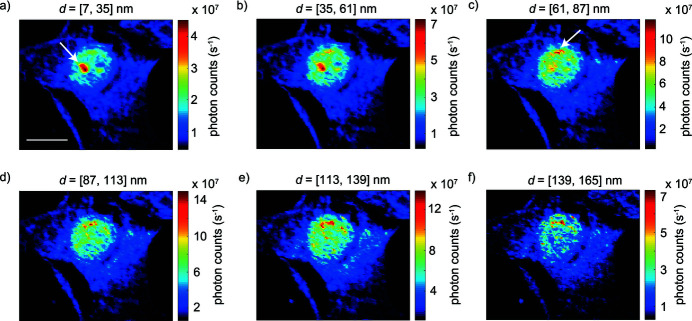
Calculated dark field images showing the granularity of the cell on different length scales. The color scale of each image ranges from the minimum to maximum number of detected photon counts for the corresponding *q*-range. See Fig. S3 for a version of this figure with the same color scale used for all subfigures. The nucleolus structure is represented by the globular region of high scattering power, as indicated by the increase in detected photon counts, on length scales of (*a*) 7 to 35 nm (white arrow) and (*b*) 35 to 61 nm. Heterochromatin is represented by regions of high scattering power at the periphery of the nucleus, and is observed on length scales of (*c*) 61 to 87 nm (white arrow), (*d*) 87 to 113 nm, (*e*) 113 to 139 nm and (*f*) 139 to 165 nm. Euchromatin fills the remainder of the nucleus on all length scales and is depicted in a light blue color. The scale bar in (*a*) is 10 µm and applies to all subfigures.

The dark field image shows an integrated quantity and reveals how much the electron density of the sample differs from the background. As it does not distinguish between length scales, to access the relevant scales within different regions of the nucleus we calculate dark field images for different *q*-ranges, as shown in Fig. 3 ▸. In this representation, the color scales of the individual dark field images differ, and account for the minimum and maximum of the total number of detected photons within the corresponding *q*-range. A version of Fig. 3 ▸, where all dark field images have the same color scale adjusted to the minimum and maximum number of photon counts found throughout all six images, is shown in Fig. S3 in the supporting information. By calculating various dark field images, we are able to spatially distinguish areas which contain structures of different Fourier components, corresponding to certain real space ranges. These ranges are chosen to correspond to the relevant length scales of the formation of chromatin and its subsequent packing.

Within the nucleus of a eukaryotic cell, 2 nm-thick DNA double helices (Watson & Crick, 1953 ▸) are wrapped around octamers of histones, forming 10 nm-diameter nucleosomes (Olins & Olins, 1974 ▸). Nucleosomes spaced along the genome form a nucleofilament, which is often referred to as ‘beads on a string’ (Olins & Olins, 1974 ▸). Within the traditional ‘hierarchical helical folding model’ (Sedat & Manuelidis, 1978 ▸), a nucleofilament is continuously packed into coils in a hierarchical manner. The first hierarchy forms a 30 nm-diameter chromatin fiber, and subsequent hierarchy levels form coils up to 700 nm in size, which eventually lead to the formation of individual chromosomes approximately 1 µm in size. The exact structure of the hierarchy levels is a topic of debate (Woodcock *et al.*, 1984 ▸; Maeshima *et al.*, 2010 ▸).

Compared with the dark field image shown in Fig. 2 ▸(*a*), all subfigures of Fig. 3 ▸ are noticeably different. One of the most pronounced features is the globular structure appearing in red and indicated by the white arrow in Fig. 3 ▸(*a*). This structure is prominently observed only for Fourier components corresponding to length scales of 7–61 nm. However, when regarding the adjusted dark field images shown in Figs. S3(*a*) and S3(*b*), it becomes apparent that the scattered intensity actually increases as structures within the 35–61 nm range are imaged. From comparison with typical visible-light phase contrast or differential interference contrast micrographs (Andersen *et al.*, 2002 ▸; Hernandez-Verdun *et al.*, 2010 ▸), we interpret this region as a nucleolus. The diameter of a nucleolus is roughly 2 µm (Andersen *et al.*, 2002 ▸), which corresponds well to the size of the intense regions in Figs. 3(*a*) and 3(*b*) ▸. A nucleolus is responsible for rRNA synthesis (Brown & Gurdon, 1964 ▸) and is composed of DNA (Ritossa & Spiegelman, 1965 ▸; Phillips *et al.*, 1971 ▸; Dekker & Steensel, 2013 ▸), RNA and several hundred types of proteins (Andersen *et al.*, 2002 ▸), including the phosphoprotein nucleolin (Tajrishi *et al.*, 2011 ▸). This protein accounts for approximately 10% of the protein content within the nucleolus and has a diameter of roughly 15 nm (Love & Walsh, 1968 ▸; Tajrishi *et al.*, 2011 ▸), corresponding to the length scales detected here.

As shown throughout Figs. 3(*b*)–3(*f*) ▸, as the probed length scales increase, the nucleolus structure becomes less pronounced while structures near the periphery of the nucleus become more prominent, as indicated by the white arrow in Fig. 3 ▸(*c*). The structures are primarily observed for Fourier components corresponding to length scales from 61 to 165 nm. When viewing the adjusted dark field images [Figs. S3(*b*)–S3(*f*)], the structures are observed throughout the 35–165 nm range. Regions of densely packed DNA that are found at the periphery of the nucleus are referred to as heterochromatin in the literature (Hernandez-Verdun *et al.*, 2010 ▸; Pueschel *et al.*, 2016 ▸). In agreement with what we observe in the dark field images of Figs. 3(*c*)–3(*f*) ▸ and Figs. S3(*c*)–S3(*f*), higher-order chromatin structures have been observed on length scales ranging from 80 to 160 nm in fixed *Drosophila melanogaster* embryonic chromosomes (Belmont *et al.*, 1989 ▸). Heterochromatin is necessary for both the expression of heterochromatic genes and the inhibition of the expression of euchromatic genes (Weiler & Wakimoto, 1995 ▸).

Throughout all length scales shown in Fig. 3 ▸, we observe structures within the nucleus in addition to the nucleolus or heterochromatin. See, for example, structures which scatter approximately 2.5 × 10^7^ photons s^−1^ in Fig. 3 ▸(*b*) and are represented by a light blue color on the corresponding color scale. Similar structures are observed for every subfigure of Fig. 3 ▸ and all have a lower scattering power compared with the heterochromatin or nucleolus, as indicated by the relatively decreased number of detected photons. The regions likely contain loosely packed DNA, referred to as euchromatin. Euchromatin is known to be gene-rich and involved in active processes such as transcription (Kwon & Workman, 2011 ▸). We observe that at length scales of 139–165 nm [Fig. 3 ▸(*f*)] euchromatin structures partially disappear. At these length scales, the transition from a smaller to larger hierarchy level may be occurring, thus the comparatively small structure sizes of loosely packed euchromatin are not as clearly visible compared with the more-condensed heterochromatin. When observing the adjusted dark field images in Fig. S3, it becomes clear that the scattered intensity from euchromatin is approximately the same for length scales within the 7–61 nm range, and increases on length scales from 61 to 139 nm. On length scales of 139–165 nm, Fig. S3(*f*) shows a decrease in intensity, similar to that observed in Fig. 3 ▸(*f*).

**Figure 4:**
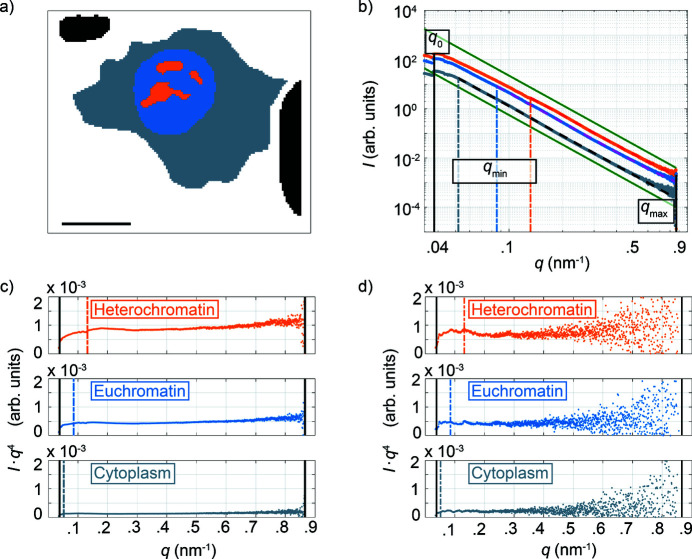
(*a*) Manually selected regions of interest. The heterochromatin and euchromatin regions are shown in orange and blue, respectively, the cytoplasm in gray and the background in black. The scale bar is 10 µm. (*b*) The scattering patterns belonging to each region are averaged, azimuthally integrated, background-corrected and plotted against the absolute value of the scattering vector *q*. The radial intensity profiles are fitted using a power law decay [equation (3)] from *q*_min_ to *q*_max_, as shown by the bold dashed lines. The vertical dashed lines represent the *q*_min_ value of the respective ROI. The solid black lines at 0.038 nm^−1^ and 0.867 nm^−1^ represent *q*_0_ and *q*_max_, respectively. The solid green lines are proportional to *q* ^−4^ and serve only as a visual aid to the overall *I*(*q*) decay. (*c*) 1D radial intensity profiles, corresponding to the averaged scattering patterns of the regions of interest, plotted as *I* *q* ^4^ versus *q* (Porod plot). The left and right vertical black lines represent *q*_0_ and *q*_max_, respectively. The vertical dashed lines represent *q*_min_. Samples that can be described using Porod’s law exhibit a characteristic plateau in the Porod plot. (*d*) Porod plots corresponding to a single, typical scattering pattern from each region of interest. *I* *q* ^4^ values below zero are omitted.

From the various dark field images shown in Fig. 3 ▸, structures are observed throughout all length scales, namely the nucleolus, heterochromatin and euchromatin. These structures are composed of material scattering with Fourier components corresponding to a size range from 7 nm to 165 nm and represent a portion of the length scales covered throughout the entire DNA packaging process, *i.e.*, 2 nm to 1 µm. Subsequent analysis is based on defining ROIs by visual inspection of a dark field image. The dark field image shown in Fig. 2 ▸(*a*) is calculated using the largest *q*-range possible, corresponding to all structures within the [7, 165] nm range, and represents the sum of all dark field images shown in Fig. 3 ▸. For this reason, Fig. 2 ▸(*a*) is used to define the ROIs. The ROIs, as shown in Fig. 4 ▸(*a*), represent euchromatin in blue and the cytoplasm in gray. A background ROI (black) is selected and used for subsequent data correction. We combine the nucleolus and heterochromatin structures into one ROI (orange, in the following referred to as heterochromatin), as a separate analysis of the two regions results in only minor differences in the values of α and *K*, see Fig. S4 in the supporting information.

To characterize each ROI, the corresponding 2D scattering patterns are averaged, subsequently azimuthally integrated and the resulting intensity values *I* are plotted with respect to the magnitude of the scattering wavevector *q*. We exploit plots of *I*(*q*) to probe structural information concerning the morphology and aggregation state of biological material inside the cells. To this end, *I*(*q*) data are background corrected and fitted using a power law (Porod, 1951 ▸; Guinier & Fournet, 1955 ▸),
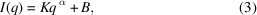
where the constant *B* accounts for inelastic and incoherent scattering. The exponent α describes the dimensionality, shape and surface roughness of the sample. For smooth, three-dimensional objects we expect α = −4, for two-dimensional objects α = −2 and for one-dimensional objects α = −1 (Glatter & Kratky, 1982 ▸). Non-integer values of α can be attributed to polydisperse samples or diffusive particle boundaries (Schmidt, 1982 ▸). In particular, diffusive boundaries can be characterized by α < −4, and polydispersity by α > −4. Note that the units of equation (3) are only well defined when the exponent is an integer. Fig. 4 ▸(*b*) shows an example of fitted *I*(*q*) curves; these curves exhibit one power-law regime. Interestingly, 24 of the 33 cells analyzed have *I*(*q*) curves which exhibit two power-law regimes with an apparent ‘kink’ around *q* ≃ 0.1 nm^−1^, where the slopes transition from higher towards lower values. Fig. S5(*c*) in the supporting information shows an example of such *I*(*q*) curves. Additionally, Figs. S2(*c*) and S2(*d*) show phase contrast micrographs of the same cell in the chemically fixed and lyophilized state, respectively. This ‘kink’ phenomenon has also been observed previously (Weinhausen *et al.*, 2014 ▸) for a different cell type.

To access local structural information, resolved in real space on the length scale of the beam size, *I*(*q*) curves belonging to individual scattering patterns are analyzed. A map of α values for each scan position, determined by using equation (3) to fit individual *I*(*q*) curves from *q*_0_ to *q*_min_, where *q*_0_ is fixed and *q*_min_ is unique for each ROI, is shown in Fig. 2 ▸(*b*). For the single cell shown in Fig. 2 ▸(*b*) the heterochromatin ROI is fitted from [*q*_0_, *q*_min_] = [0.038, 0.132] nm^−1^, relevant to structures with length scales of [48, 165] nm in real space, and a median value of α = −3.5 is found. Similarly, the euchromatin ROI is fitted from [0.038, 0.085] nm^−1^, relevant to length scales of [74, 165] nm in real space, and a median value of α = −3.4 is found. The median values indicate that the material distributed within the heterochromatin and euchromatin ROIs have similar local morphology. In the map shown in Fig. 2 ▸(*b*) one globular region resembling a nucleolus is faintly observed, as indicated by the white arrow. Compared with the surrounding DNA, the nucleolus shows increased α values, indicating that it has some degree of morphological difference compared with neighboring structures. This difference could be associated with the various proteins known to compose the nucleolus. Within the cytoplasm of the cell shown in Fig. 2 ▸(*b*) we find very noisy values, which is most likely explained by the small [*q*_0_, *q*_min_] = [0.038, 0.052] nm^−1^ range, corresponding to only 32 data points, fitted for this particular cell. For the *N* = 33 cells, the average [*q*_0_, *q*_min_] range fitted for the cytoplasm ROIs is [0.038, 0.084] nm^−1^, corresponding to 86 data points.

Even though equation (3) may be used to characterize data in the case of α ≠ −4, the equation is only referred to as Porod’s law (Porod, 1951 ▸; Glatter & Kratky, 1982 ▸) in the special case of α = −4. To determine the *q*-range in which α = −4, we systematically, and for each ROI of every cell, separately fit the averaged *I*(*q*) curve with different *q*-ranges using equation (3) and compare the fit coefficients for α with the theoretical value of −4. The largest *q*-range which obeys the enforced threshold condition (see *Materials and methods* [Sec sec2]) defines [*q*_min_, *q*_max_]. Alternatively, instead of plotting the intensity *I* versus the scattering vector *q* to determine [*q*_min_, *q*_max_], it is possible to use so-called Porod plots (Ciccariello *et al.*, 1988 ▸) of *I* *q* ^4^ versus *q*. In this representation, a characteristic plateau appears in the range [*q*_min_, *q*_max_]. Porod plots of data averaged over each ROI are shown in Fig. 4 ▸(*c*), and typical individual data sets from each ROI are shown in Fig. 4 ▸(*d*).

When comparing our data with the literature, we obtain a consistent picture. For lyophilized samples, values of α ≃ −4 are typically reported (Weinhausen *et al.*, 2012 ▸; Hémonnot *et al.*, 2016 ▸; Nicolas *et al.*, 2017 ▸; Bernhardt *et al.*, 2018 ▸). However, previous work (Weinhausen *et al.*, 2014 ▸) comparing chemically fixed-hydrated cells and living cells has shown that the sample preparation procedure has a considerable influence on measured values of α. Systematically higher values of α, *i.e.*, closer to zero, for both sample types are reported and values in the range −4.0 < α < −3.0 and −3.0 < α < −2.5, for chemically fixed and living cells, respectively, were found.

If α is fixed at −4, *K* [equation (3)] is termed the Porod constant and depends on the electron density contrast 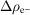 of the sample and the surface area *S* of the interface between scatterers and environment (Guinier & Fournet, 1955 ▸; Glatter & Kratky, 1982 ▸),
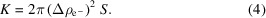
Equation (4) assumes that both the sample and background are composed of homogeneous electron densities where 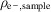 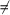 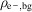. Thus, it is not directly applicable to biological samples but serves as an aid to understanding the relationship between 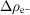 and *S*. A large Porod constant value can be due to (i) a large interface area *S*, (ii) a large electron density contrast 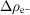 between the two phases of the sample or (iii) a combination of both. Therefore, the Porod constant *K* is used as an aid to understand the aggregation state of the scatterers. A map of *K*, derived by using equation (3) to fit individual *I*(*q*) profiles within [*q*_min_, *q*_max_], where α = −4, is shown in Fig. 2 ▸(*c*). The DNA distribution at the periphery of the nucleus, as indicated by the red arrow in Fig. 2 ▸(*c*), is mostly in agreement with the heterochromatin distribution observed throughout the dark field images shown in Figs. 3(*b*)–3(*f*) ▸ and Figs. S3(*b*)–S3(*f*). As heterochromatin is tightly packed chromatin, resulting in a larger electron density compared with euchromatin, it is reasonable to expect the heterochromatin distributions in the Porod constant map and dark field images to resemble each other. In agreement with the dark field images shown in Figs. 3(*a*) and 3(*b*) ▸ and Figs. S3(*a*) and S3(*b*), a globular region resembling a nucleolus is distinguishable in the *K* map, as indicated by the white arrow in Fig. 2 ▸(*c*). Since *K* is only linearly proportional to *S* but proportional to the square of 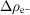, and a nucleolus is densely filled with proteins, we expect to observe the nucleolus in the *K* map. However, it is additionally possible that the total interface area *S* of the material within the nucleolus is large.

### 3.2. Quantitative density measurements in the cell nucleus   

From the discussion above it becomes clear that scanning SAXS measurements provide information about the morphology and aggregation state of nuclear material; however, the electron density 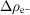 is not quantitatively accessible. Thus, we combine the scanning SAXS measurements with X-ray in-line holography to access both the projected electron density and projected mass density. In contrast to scanning SAXS, which is sensitive to structures of typical length scales, holography is a full-field imaging technique sensitive to the integrated electron density of material along the propagation direction of the X-rays.

In the holography setup, shown schematically in Fig. 1 ▸(*b*), the KB-focused X-rays are coupled into a waveguide (Chen *et al.*, 2015 ▸) that acts as a quasi point source (≤20 nm), emitting a highly divergent wavefront which is ideally suited for near-field imaging. Compared with the KB-focused beam the waveguide increases the numerical aperture of the system, thus increasing the imaging resolution. The sample is placed at a series of defocused positions, *X*_1_, and full-field holograms are recorded at a sample-to-detector distance of *X*_2_. Holograms are recorded at multiple distances to account for the zero-crossings of the phase contrast transfer function produced when imaging weakly absorbing objects with a slowly varying phase, *e.g.*, biological samples (Zabler *et al.*, 2005 ▸). By adjusting the geometric magnification of the system, given by 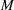 = 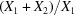, the FOV can be tailored to accommodate the imaging of either a single or a group of multiple cells. Thereby, the effective pixel size of the holograms, given by 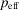 = 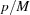, where *p* is the pixel size of the detector, is varied.

We initiate the phase reconstruction process by numerically processing the acquired holograms *via* the CTF algorithm. The resulting 2D reconstructed phase map, denoted as −ϕ(*x*, *y*), is then employed to define the support constraint used in the RAAR algorithm. In short, one iteration of the reconstruction process begins by propagating the measured intensity from the detector plane to the object (sample) plane. An object support is subsequently applied, which accounts for the shape of a spatially resolvable object. Here, the phase map rendered from the CTF reconstruction is used to define the object support and is chosen to account for both the cell body as well as its surrounding background. The waveform is then propagated towards the detector plane, where its amplitude is replaced by the square-root of the measured intensity, *i.e.*, the modulus constraint, and is subsequently propagated back to the object plane. The iteration round is now complete. This waveform then serves as the starting point for the next iteration. The object support is held constant throughout the entire iterative process. After the phase reconstruction process is complete, the phase contribution of the cell itself is determined. The median value of the background region, shown in black in Fig. 4 ▸(*a*), is determined and subtracted from the rest of the reconstructed phase map, *i.e.*, ϕ(*x*, *y*) = ϕ_sample_ − ϕ_bg_; the resulting values of ϕ(*x*, *y*) are used for subsequent analysis.

The reconstructed phase is directly related to the 2D projected electron density (electron density per area) (Cloetens *et al.*, 1999 ▸), 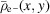, *via*

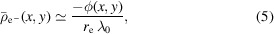
where *r*_e_ and λ_0_ denote the classical electron radius and the X-ray incident wavelength, respectively. The projected electron density is related to the 2D projected mass density (Giewekemeyer *et al.*, 2010 ▸; Wilke *et al.*, 2015 ▸), 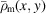, *via*

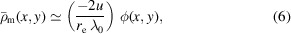
where *u* is the atomic mass unit. The factor of 2 is dependent on the chemical composition of the sample. For cellular constituents with an average empirical formula of H_50_C_30_N_9_O_10_S_1_ the factor of 2 is valid (Giewekemeyer *et al.*, 2010 ▸).

To correlate the holographic and scanning SAXS data in a pixel-wise manner, all resulting maps from the holography and scanning SAXS measurements must have the same pixel size and FOV. To this end, we register the reconstructed phase maps to the dark field images, thus rendering them suitable for direct comparison. An example of a registered 2D reconstructed phase map is shown in Fig. 2 ▸(*d*). The nucleus and cell body are clearly distinguishable. In particular, two globular regions are observed, as indicated by the black arrows. This phase map is reconstructed using holograms recorded at a single distance; the recording of holograms at multiple distances is performed to ensure the optimal phase retrieval for the CTF algorithm, which subsequently serves as the support constraint for the single distance RAAR algorithm.

As ϕ, 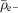 and 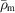 are proportional to each other, Fig. 2 ▸(*d*), in addition to the measured phase shift, also shows the 2D projected electron and projected mass density maps, see scale bars on the right- and left-hand side, respectively. Overall, we find median values of 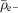 for the heterochromatin, including the nucleoli, and euchromatin regions to be 2.1 × 10^19^ e^−^ cm^−2^ and 1.1 × 10^19^ e^−^ cm^−2^, with corresponding 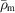 values of 0.07 and 0.04 mg cm^−2^, respectively. Within the cytoplasm, we find median values of 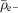 = 5.1 × 10^18^ e^−^ cm^−2^ and 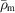 = 0.02 mg cm^−2^. The reported values of the projected mass density are comparable with lyophilized *Deinococcus radiodurans* (Giewekemeyer *et al.*, 2010 ▸) and lyophilized bacterial endospores (Wilke *et al.*, 2015 ▸).

In our holography setup the accessed momentum transfer *q* is shifted towards smaller values, *i.e.*, larger real space structures, compared with the scanning SAXS experimental setup, which has a similar sample-to-detector distance. To estimate the resolution of the reconstructed phase map shown in Fig. 2 ▸(*d*), the power spectral density (PSD) is calculated and azimuthally averaged. The resolution is approximated by the transition from signal to noise and, for our experimental setup, is found around *q* = 0.119 nm^−1^, corresponding to a real space resolution of 53 nm. We can thus conclude that the DNA structures observed in the projected electron density map correspond to length scales of at least 53 nm. Fig. S6 in the supporting information shows a comparison between the PSD and a typical *I*(*q*) profile derived from the scanning SAXS measurements. We observe the overall slope of the PSD to be ∼−3, in contrast with the *I*(*q*) slope of ∼−4. As also shown in Fig. S6, the combination of holography and scanning SAXS enables access to *q*-values spanning three orders of magnitude (Nicolas *et al.*, 2017 ▸). However, as a direct consequence of the different *q*-ranges accessed by each imaging modality with only little overlap, we cannot directly combine the data and quantify of the interface area *S via* equation (4) by inserting 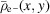.

Holography is sensitive to the collective electron density of the probed material, thus indicating that the two globular regions in the projected electron density map shown in Fig. 2 ▸(*d*) are dense regions. In agreement with visible-light phase contrast images (Andersen *et al.*, 2002 ▸; Hernandez-Verdun *et al.*, 2010 ▸) and our interpretation from the dark field images shown in Figs. 3(*a*) and 3(*b*) ▸ and Figs. S3(*a*) and S3(*b*), we suggest that these regions are nucleoli. In contrast to the projected electron density map, where two nucleoli are observed, only one nucleolus is prominently observed in the Porod constant *K* map [Fig. 2 ▸(*c*)]. Interestingly, only the left nucleolus is pronounced in both maps; the right nucleolus is less pronounced in the *K* map. The most likely explanation for the variation between the two maps is that the local material in the right nucleolus is composed of dense material that is aggregated into a volume with a small interface area *S*. According to equation (4), a small interface area would decrease *K*.

### 3.3. DNA aggregation and density for a cell ensemble   

**Figure 5:**
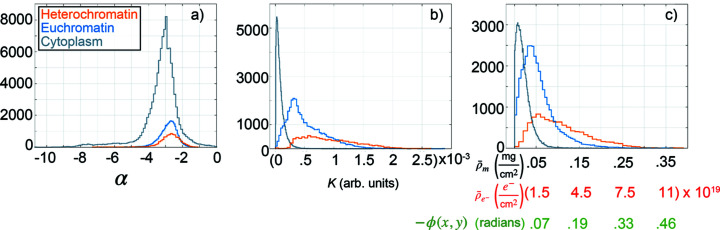
Histograms of variables from fitting individual *I*(*q*) profiles of *N* = 33 cells. The *y*-axes represent the number of counts. The heterochromatin and euchromatin distributions are shown in orange and blue, respectively. The cytoplasm distributions are gray. (*a*) The exponent α, equation (3), from fitting *I*(*q*) profiles in the [*q*_0_, *q*_min_] range where α ≠ −4. (*b*) The Porod constant *K*, derived from fitting profiles in the [*q*_min_, *q*_max_] range where α = −4. (*c*) The projected mass density (black axis labels), projected electron density (red axis labels) and phase shift (green axis labels). The number of bins is calculated using Scott’s rule (Scott, 1979 ▸). The median values of each distribution are listed in Table 1 ▸.

So far, we have discussed typical results for a particular cell that we investigated in this study. The same analysis was performed for an ensemble of *N* = 33 cells. Fig. 5 ▸ shows distributions of all variables discussed above for all measured positions within all cells, separately for the cytoplasm and the two nuclear regions, thus adding statistical relevance to our results. A total of 10425 scattering patterns were analyzed for the heterochromatin region, 36655 for the euchromatin region and 106460 for the cytoplasm. The median values of the distributions are listed in Table 1 ▸. As shown in Fig. 5 ▸(*a*), for data that do not obey Porod’s law, *i.e.*, data within [*q*_0_, *q*_min_] where α ≠ −4, we find similar median values for α throughout the three ROIs, albeit slightly smaller for the cytoplasm regions. Two-component Kolmogorov–Smirnov (Kolmogorov, 1933 ▸) (KS) tests were performed with a null hypothesis that the three distributions shown in Fig. 5 ▸(*a*) do not significantly differ. At the 5% significance level the null hypothesis is rejected, indicating all three distributions are significantly different from one another.

The distributions of the Porod constant *K*, derived by fitting data within [*q*_min_, *q*_max_] where α = −4, are shown in Fig. 5 ▸(*b*). We find that for each ROI the distribution of *K* values varies, similar to the map shown in Fig. 2 ▸(*c*). The largest median value of *K* is reported for the heterochromatin regions of the cells (orange), indicating that the product of the interface area *S* and projected electron density contrast squared is a factor of 1.9 larger than for the euchromatin regions (blue) and 13.7 larger than for the cytoplasm regions (gray).

As the reconstructed phase ϕ, projected electron density 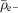 and projected mass density 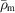 are all directly related to one another, differing only by constant factors [see equations (5) and (6)], Fig. 5 ▸(*c*) shows the distributions of all three variables. We find that within the heterochromatin ROIs of all 33 cells there is a factor of 1.8 more material than within the euchromatin regions, as described by the relative increase in both the projected electron density and projected mass density. Our calculated factor of 1.8 is on the same order of magnitude as in the confocal microscopy study presented by Sadoni *et al.* (2001 ▸), who concluded that heterochromatin of living HeLa cells is a factor of 1.4 as condensed as euchromatin. Compared with the cytoplasm regions, we find the heterochromatin regions to contain a factor of 4.5 more material.

## 4. Summary and conclusions   

To summarize, we exploit the short wavelength and high penetration depth of X-rays to image whole, intact cells. In particular, we combine X-ray holography and scanning SAXS, including the analysis of dark field representations and the power law fits of intensity profiles, *I*(*q*). By this threefold view on a complex biological system, the cell nucleus, we obtain access to the projected electron and mass densities, length scales of the scatterers, and aggregation and morphology of nuclear material. Separate dark field representations for different ranges of momentum transfer reveal nuclear regions containing nucleoli, heterochromatin or euchromatin, respectively, as the method is highly sensitive to the prominent length scale of the scatterers. Analysis of the power law exponent α, which gives rise to differences in scatterer morphology, cannot clearly distinguish the different nuclear regions, which is typical for complex biological matter. By contrast, the Porod constant *K*, a measure for the aggregation of the scatterers, reveals regions dominated by heterochromatin and the locations of some, but not all, nucleoli. Finally, X-ray holography is able to clearly distinguish the nucleoli by quantitative electron and mass density analysis, see summary in Table 2 ▸.

Turning this line of arguments around, for the methods we employed here, comparatively loosely packed euchromatin is visible only in the scaled dark field representations, denser, more aggregated heterochromatin in dark field and *K* maps, and, finally, the very dense, strongly aggregated nucleoli in all three analyses. From a biological point of view, we find that nucleoli are the densest structures in the nucleus, scattering mostly on length scales up to about 60 nm, indicating the existence of structures in this size range, possibly proteins. While the density of heterochromatin and euchromatin is similar and lower than for nucleoli, as revealed by X-ray holography, heterochromatin mostly scatters on length scales above 35 nm and euchromatin scatters on all probed length scales. The Porod constant reveals that, compared with euchromatin, heterochromatin and nucleoli are more aggregated. In agreement with the literature (Belmont *et al.*, 1989 ▸; Hernandez-Verdun *et al.*, 2010 ▸; Pueschel *et al.*, 2016 ▸), we find heterochromatin to be located near the periphery of the nucleus and euchromatin to fill all other regions that are not occupied by heterochromatin or the nucleoli. Our study highlights the importance of combined imaging approaches that capture multiple length scales for the characterization of complex biological systems. The combination of scanning SAXS and X-ray holography is straightforward and can be realized in a single experimental setup and can easily be applied to other biological systems.

## Supplementary Material

Sections S1 to S6 and Figures S1 to S6. DOI: 10.1107/S1600577520016276/ju5016sup1.pdf

## Figures and Tables

**Table 1:** Median values of the distributions shown in Fig. 5 ▸; the values of α and *K* are derived by fitting *I*(*q*) profiles between [*q*_0_, *q*_min_] and [*q*_min_, *q*_max_], respectively

Region	α	*K* (a.u.)	ϕ(*x*,*y*) (rad)	\bar{\rho}_{{\rm{e}}^{-}} (e^−^cm^−2^)	\bar{\rho}_{\rm{m}} (mg cm^−2^)
Heterochromatin	−2.68	8.35 × 10^−4^	−0.13	2.85 × 10^19^	0.09
Euchromatin	−2.78	4.31 × 10^−4^	−0.06	1.36 × 10^19^	0.05
Cytoplasm	−3.09	6.10 × 10^−5^	−0.02	5.00 × 10^18^	0.02

**Table 2:** Summary of the parameters probed by combined scanning SAXS and X-ray holography, including the nuclear structures accessible by each of the analyses and the physical property probed by the respective method

Analysis approach	Nuclear structures accessed	Physical property probed
Dark field	Nucleoli, heterochromatin, euchromatin	Total scattering, Fourier components relevant for specific length scales
Power law exponent α	(Nucleoli)	Morphology
Porod constant *K*	Nucleoli, heterochromatin, euchromatin	Aggregation state, interface area
Phase shift	Nucleoli	Electron/mass density
