# Supplementary material for: Combined scanning small-angle X-ray scattering and holography probes multiple length scales in cell nuclei
Source: J Synchrotron Radiat. 2021 Jan 21;28(Pt 2):518–29. doi: 10.1107/S1600577520016276 (PMC7941289; doi:10.1107/S1600577520016276)
Supplement: Supplementary file 1 [file s-28-00518-sup1.pdf]

Supporting Information for: Combined Scanning Small Angle X-Ray Scattering and Holography Probes Multiple Length Scales in Cell Nuclei

Andrew Wittmeier[a], Chiara Cassini[a,b], Mareike Töpperwien[a], Manuela Denz[a], Johannes Hagemann[a,c], Markus Osterhoff[a], Tim Salditt[a,b], and Sarah Köster[a,b,*]

[a]Institute for X-Ray Physics, University of Göttingen, Friedrich-Hund-Platz 1, 37077 Göttingen, Germany
[b]Cluster of Excellence "Multiscale Bioimaging: from Molecular Machines to Networks of Excitable Cells" (MBExC), University of Göttingen, Germany
[c]Current address: Deutsches Elektronen-Synchrotron, Notkestraße 85, 22607 Hamburg, Germany
[*]sarah.koester@phys.uni-goettingen.de

S1 Background correction of radial intensity profiles

The data of all scanning SAXS measurements were background corrected using the radial intensity profiles $I(q)$. For every cell, the $I(q)$ profile of each region was obtained by azimuthally integrating the average diffraction pattern of that region. The profile was then corrected by subtracting the $I(q)$ profile corresponding to the background region. See Figure S1.

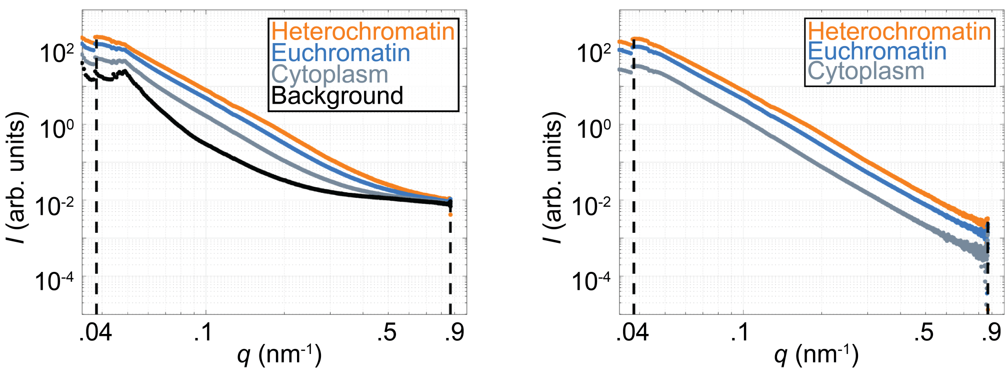

Figure S1: Left: Radial intensity profiles for the heterochromatin, euchromatin, cytoplasm and background ROIs shown in Figure 4a of the main text. The profiles were obtained by azimuthally integrating the average diffraction pattern belonging to each region. The vertical dashed lines at $q = 0.038$ nm^{-1} and $q = 0.867$ nm^{-1} represent q_0 and q_{max}, respectively. Right: Radial intensity profiles after background subtraction.

S2 Visible light phase contrast microscopy

Figure S2 shows visible light phase contrast images of chemically-fixed and lyophilized cells adhered to Si_3N_4 membranes. Images were acquired using an inverted microscope (IX81, Olympus, Hamburg, Germany) equipped with a Retiga 6000 Monochrome camera (QImaging, Tucson, AZ, USA) and a 20× objective (UCPlanFLN, N.A.= 0.7; Olympus).

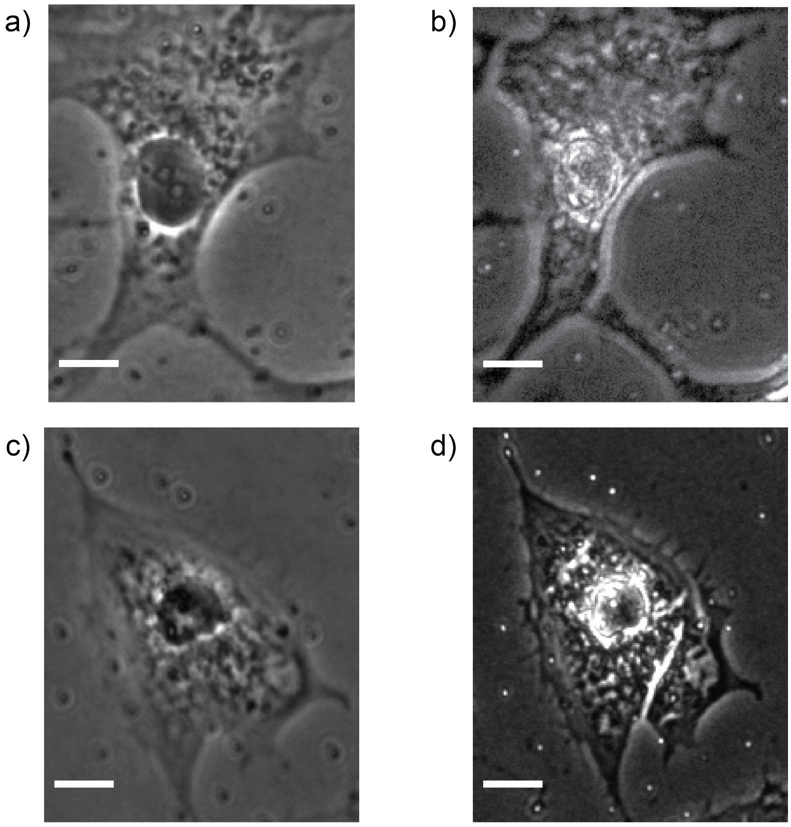

Figure S2: Visible light phase contrast images of NIH-3T3 fibroblasts adhered to Si_3N_4 membranes. The cell shown throughout the main text in the a) chemically-fixed and b) lyophilized state. The cell shown in Figure S5 in the c) chemically-fixed and d) lyophilized state. All scale bars are 10 µm.

S3 Dark field comparison

Figure S3 shows the same dark field images shown in Figure 3 of the main text with adjusted color scales. All subfigures in Figure S3 have the same color scale adjusted to account for the minimum and maximum number of detected photons throughout all subfigures.

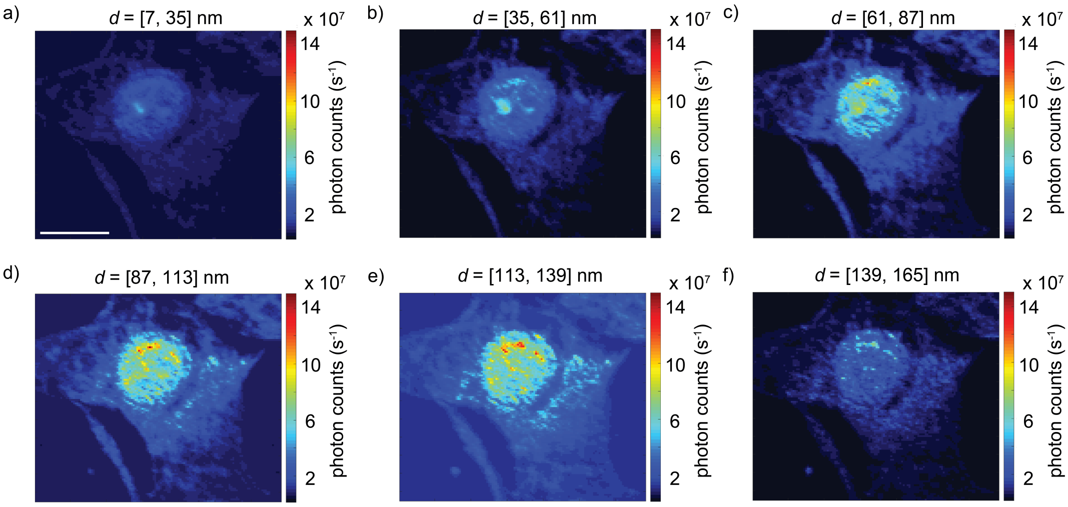

Figure S3: Dark field images of the cell shown in Figure 3 of the main text. Here, all images have the same color scale. Compared to all other subfigures, the contrast of subfigure e) is lower due to high-intensity streaks of the primary X-ray beam localized within the corresponding q-range. The scale bar is 10 µm and applies to all subfigures.

S4 Nucleoli Region of Interest

In the main text the "heterochromatin ROI" contains the heterochromatin and nucleoli structures. To justify this combination, a ROI of only the nucleoli is created and analyses of the heterochromatin ROI with and without the nucleoli ROI included are compared. The diffraction patterns belonging to each ROI are averaged and azimuthally integrated to obtain 1D $I(q)$ profiles. The exponent α, derived by fitting $I(q)$ in the $[q_0, q_{\text{min}}]$ range, is determined. For the heterochromatin ROI with and without the nucleoli included, $[q_0, q_{\text{min}}] = [0.038, 0.132]$ nm^{-1} and $[0.038, 0.079]$ nm^{-1}, respectively, and values of -3.5 and -3.6 are found. The Porod constant K is determined by fitting in the range of $[q_{\text{min}}, q_{\text{max}}]$, where the Porod exponent $\alpha = -4$ is found. For the heterochromatin ROI with and without the nucleoli included, $[q_{\text{min}}, q_{\text{max}}] = [0.132, 0.867]$ nm^{-1} and $[0.079, 0.867]$ nm^{-1}, respectively, and values of 7.6×10^{-4} and 7.1×10^{-4} are found. Fits of the $I(q)$ profiles are shown in Figure S4c.

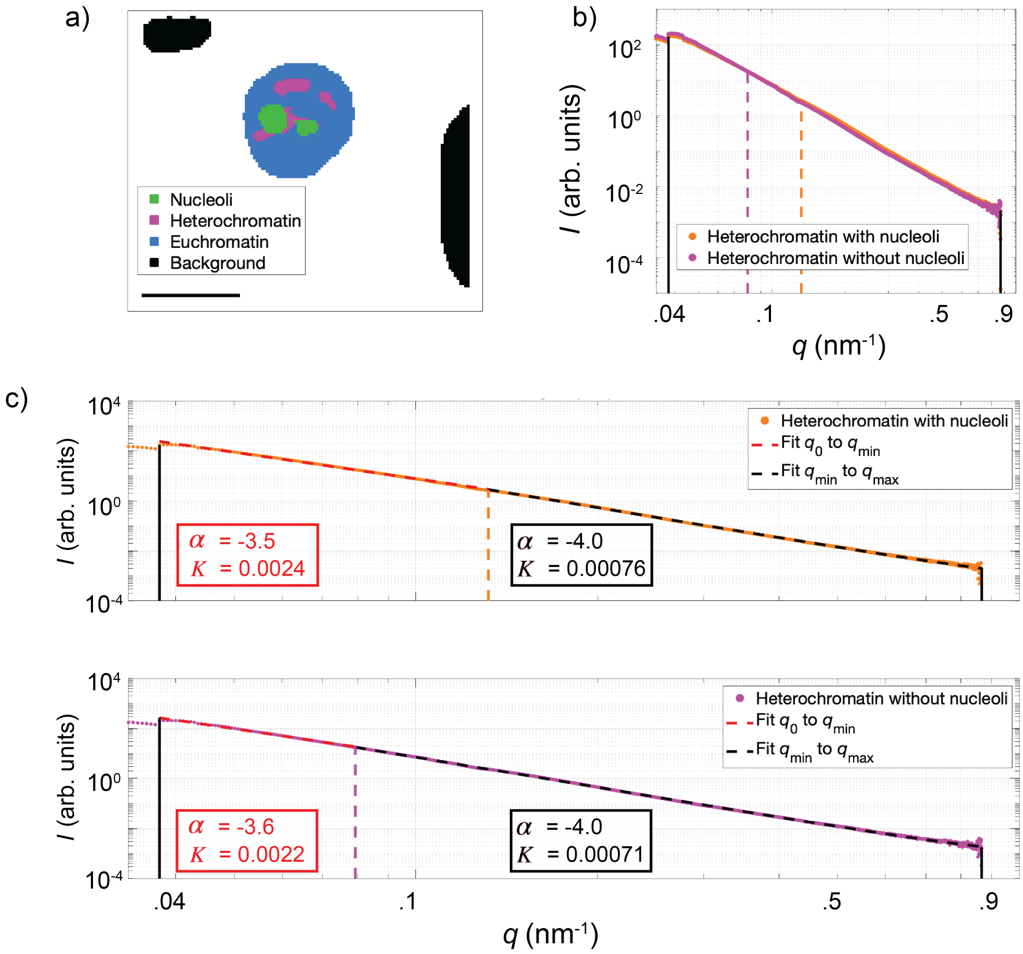

Figure S4: a) ROIs selected from the reconstructed phase map in Figure 4d of the main text. The heterochromatin ROI (purple) does not include any of the nuclei ROI (green). b) Corresponding background-corrected radial intensity $I(q)$ profiles. The purple line corresponds to the heterochromatin ROI shown in a). The orange line depicts the heterochromatin $I(q)$ profile shown in Figure 4b of the main text and corresponds to the combined heterochromatin and nucleoli ROIs. The vertical dashed lines represent $q_{\min}$ for the respective ROI. The solid, black vertical lines at $q = 0.038$ nm^{-1} and $q = 0.867$ nm^{-1} represent q_0 and $q_{\max}$, respectively. c) Fitted $I(q)$ profiles. The red and black dashed lines represent the fits of $I(q) = Kq^{\alpha} + B$ for the $[q_0, q_{\min}]$ and $[q_{\min}, q_{\max}]$ ranges, respectively.

S5 Supplementary Cell

Figure S5 is similar to both Figure 2 and Figure 4a,b of the main text and represents a different example cell. This cell exhibits a distinguishable "kink" around $q \simeq 0.1$ nm^{-1} in its $I(q)$ profiles. The dark field image calculated using the entire usable data range $[q_0, q_{\mathrm{max}}] = [0.038, 0.867]$ nm^{-1}, which corresponds to $[7, 165]$ nm in real space is shown. Additionally, the corresponding manually selected regions of interest, averaged and background corrected radial intensity $I(q)$ profiles, the exponent α, the Porod constant K, and the reconstructed phase, projected mass and projected electron densities are presented.

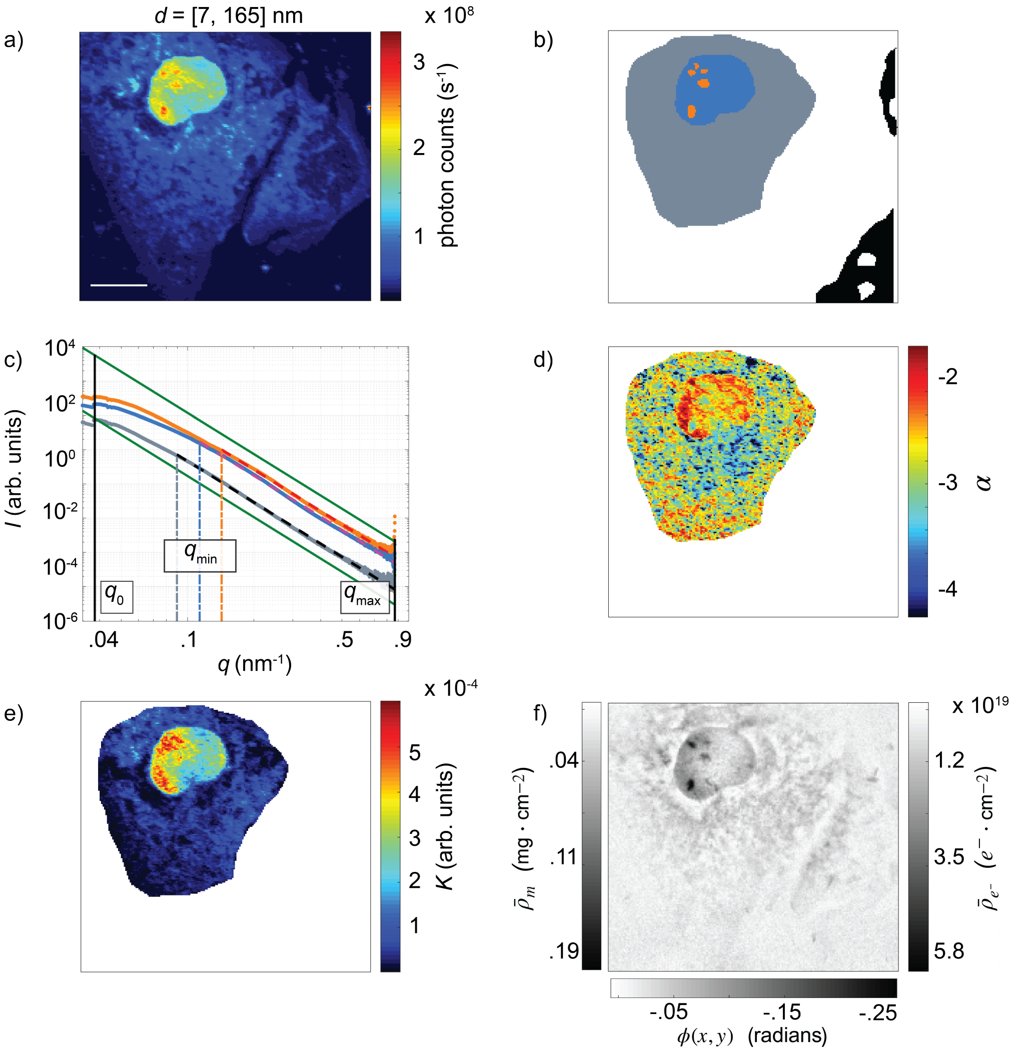

Figure S5: Additional example of a cell included in the study. (a) Dark field image for the $[q_0, q_{\max}]$ range. The color scale is adjusted for the minimum and maximum number of detected photons. The scale bar is 10 µm and also applies to subfigures (b), (d), (e) and (f). (b) Manually selected regions of interest of the heterochromatin (orange), euchromatin (blue), cytoplasm (gray) and background (black). (c) Averaged, azimuthally integrated and background-corrected diffraction patterns are plotted against the absolute value of the scattering vector q. The bold dashed lines are the fits using $I(q) = Kq^{\alpha} + B$. Around $q \simeq 0.1$ nm^{-1}, a kink becomes distinguishable between low and high q-values. The black vertical lines at $q = 0.038$ nm^{-1} and $q = 0.867$ nm^{-1} represent q_0 and $q_{\max}$, respectively. The vertical dashed lines represent $q_{\min}$ of the corresponding ROI. The solid green lines are proportional to q^{-4} and only serve as a visual aid to the overall $I(q)$ decay. (d) Map of the exponent derived by fitting $I(q)$ in the $[q_0, q_{\min}]$ range. (e) Map of the Porod constant K, derived by fitting $I(q)$ in the $[q_{\min}, q_{\max}]$ range. (f) Map of the reconstructed phase, projected mass density and projected electron density.

S6 Comparison between momentum transfers

Figure S6 shows the accessed q-range of the holographic (red) and scanning SAXS (magenta, blue) imaging modalities. The data correspond to the cell shown in Figure 2 of the main text. As the accessed q-range of each modality is different, the substitution of $\Delta\rho_{e^-}$ into $K = 2\pi(\Delta\rho_{e^-})^2 S$ to quantify the interface area between the sample and surrounding environment, S, is not possible.

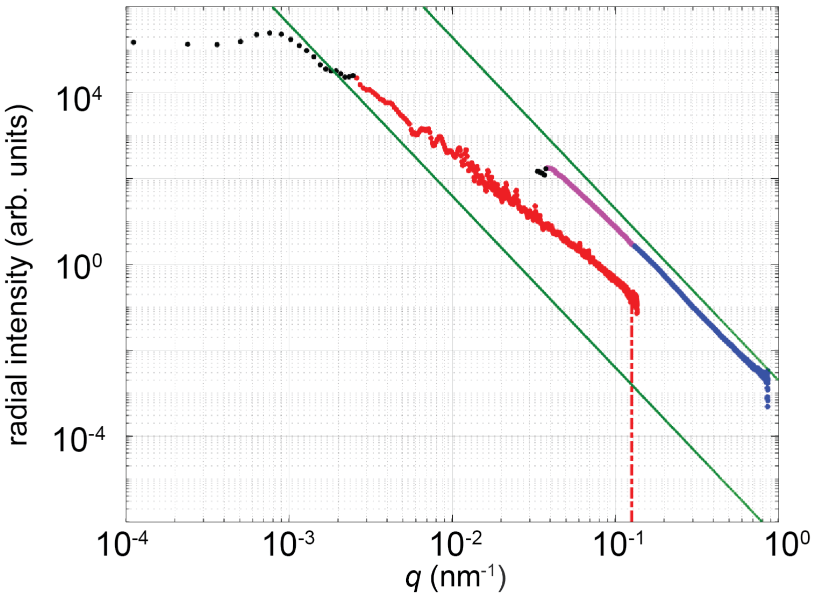

Figure S6: Comparison between the 1D averaged azimuthally integrated power spectral density (red) of a reconstructed phase map (single-distance, RAAR) and a typical radial intensity profile (magenta, blue) from scanning SAXS. The red, dashed vertical line at $q = 0.119\ \text{nm}^{-1}$ represents the transition from signal to noise and corresponds to a real-space resolution of 53 nm. The typical fitting range considered for the Porod analysis is shown in blue. The green lines are proportional to q^{-4} and serve as a visual aid to the overall power decay. Structures greater than 2.5 µm in the holography PSD are omitted (black dots). Regions blocked by the beamstop in the scanning SAXS radial intensity profile are omitted (black dots).
